# Achievement Emotions in Selective Schools: Reexamining the Happy-Fish-Little-Pond Effect in an Extreme Case from the Chinese Collectivist Context

**DOI:** 10.3390/ijerph192215399

**Published:** 2022-11-21

**Authors:** Yushi Mai, Xitong Huang, Yingting Su, Ruixiang Gao, Lei Mo

**Affiliations:** 1 Key Laboratory of Brain, Cognition and Education Sciences (South China Normal University), Ministry of Education, Guangzhou 510631, China; 2 School of Psychology, South China Normal University, Guangzhou 510631, China; 3 Center for Studies of Psychological Application, South China Normal University, Guangzhou 510631, China; 4 Guangdong Key Laboratory of Mental Health and Cognitive Science, South China Normal University, Guangzhou 510631, China; 5 Chang’an Town Zhongshan Primary School, Dongguan 523868, China; 6 School of Mathematics (Zhuhai), Sun Yat-Sen University, Zhuhai 519080, China

**Keywords:** achievement emotions, big-fish-little-pond effect, happy-fish-little-pond effect, collectivist culture, moderated serial mediation model

## Abstract

Achievement emotions, defined as the emotions generated in the academic process or by achievement results, are critical for an individual’s mental health, personality development, and academic productivity. Referring to the well-known big-fish-little-pond effect on academic self-concept, which describes the well-known phenomenon that students in selective schools/classes tend to have lower academic self-concepts than those who are comparably competent but attend regular schools/classes, Pekrun and colleagues focused on German students and proposed a similar happy-fish-little-pond effect on achievement emotions in 2019. In our paper, we examined whether this effect exists in extreme cases. To maximize the positive reflected-glory effect of being in a selective school and minimize the negative social comparison contrast effects that result from being ranked low in the school, we conducted an investigation in the Chinese collectivist cultural setting and compared the achievement emotions of students from a highly selective senior middle school with those of students from a regular school where the top-ranking students fell short of the bottom-ranking students in the selective school in terms of academic performance. Through an analysis of variance and a moderated serial mediation model, our study revealed that the bottom-ranking students in the selective school had less positive achievement emotions, lower academic self-concepts, and more negative achievement emotions than the top-ranking students in the regular school, providing strong evidence that students rely more on social comparison than on objective self-evaluation standards to evaluate themselves. The implications of the results for educational policies are discussed.

## 1. Introduction

First coined by Pekrun and his colleagues [1], achievement emotions are defined as the various emotions generated in the academic process or by achievement results, including pleasure, expectation, pride, relaxation, anger, anxiety, shame, hopelessness, and boredom. Later, the Achievement Emotions Questionnaire (AEQ) was developed, containing 24 scales that separately measure enjoyment, hope, pride, relief, anger, anxiety, shame, hopelessness, and boredom during class, while studying, and when taking tests and exams [2]. In recent years, increasing research attention has been paid to the role of emotions in education. Many studies showed that achievement emotions and academic performance influence each other. Indeed, the more individuals experience positive achievement emotions in the learning process, the better their academic performance [1]. Positive achievement emotions can not only directly help students carry out cognitive activities more smoothly [3] but also indirectly influence their academic achievement through a series of mediating variables, such as learning motivation, achievement goal, academic participation, academic efficacy, and regulation strategy [1,3,4,5,6,7]. Moreover, achievement emotions can influence school/class climate [8] and teacher–student relationships [3]. In addition to academic learning, achievement emotions play a key role in an individual’s mental health and personality development [4,8]. However, success or failure in academic performance can shape an individual’s tendency to experience achievement emotions by shaping their perceptions of their academic abilities (i.e., academic self-concepts) and expectations of failure [9,10]. In addition, other group-level or environmental factors such as learning atmosphere [11], achievement level [12], and student composition [13] in the school can affect an individual’s achievement emotions.

In 2019, Pekrun and his colleagues [14] investigated German junior high school students and proposed a “happy-fish-little-pond effect” (hereafter “HFLPE”) on achievement emotions; that is, high achievers are happier being a fish in a little pond than in a big pond. The HFLPE is consistent with the well-known big-fish-little-pond effect (hereafter “BFLPE”) on academic self-concepts proposed by Marsh and his colleagues [15]. The BFLPE describes the well-known phenomenon that students in selective schools/classes (big ponds) tend to have lower academic self-concepts than those who are comparably competent but attend regular schools/classes (little ponds), and this effect was shown to be stable and intercultural using considerable evidence [16] and meta-analyses [17]. The mechanism at the heart of these two effects lies in social comparison [18]. In addition to using objective, physical, and non-social criteria to evaluate their performance, individuals evaluate themselves by comparing themselves with others [19]. To some extent, feedback from the outside world is more important than objective self-evaluation standards [20]. At school, peers who pursue similar or common goals are the main objects of comparison [21]. According to the frame-of-reference effect, students’ academic self-concepts and achievement emotions depend not only on their absolute academic level but also on their level relative to other students in the same school/class [22]. In selective schools/classes, being ranked low results in low academic self-concepts and more negative achievement emotions in students.

However, the effect of selective schools on students’ academic self-concepts and achievement emotions may be two-sided. While being ranked low in selective schools undermines students’ academic confidence and well-being, many of the hallmark advantages of selective schools, such as good reputation, good disciplinary climate, quality teaching staff, superior socioeconomic context [23], and group effect of excellent peers [24], play a positive role. For example, the academic self-concepts of students in selective schools may be reinforced by their identity as members of an excellent group, and this reflected glory or assimilation effect may be stronger in a collectivist culture than in individualistic culture because students of a collectivist culture have stronger group consciousness and tend to use the similarity strategy rather than the contrast strategy for self-evaluation, thinking that they are as excellent as their peers [25]. Several studies demonstrated that higher perceived school status promotes students’ academic self-concepts [25,26], and thus, enables them to have a higher sense of school belonging and to generate more positive and stable achievement emotions [27,28]; nevertheless, the positive reflected-glory effect was shown to be weaker and unable to offset the negative BFLPE on academic self-concepts; that is, the net effect of selective schools on students’ academic self-concepts remains negative, although weaker, even in regions characterized by their collectivist culture, such as in Hong Kong and Chinese Taiwan [25,26,29].

Although the BFLPE on academic self-concepts has been examined in collectivist cultures, no research to date has directly investigated the HFLPE on achievement emotions in collectivist contexts. As research on academic self-concepts has indicated that the negative BFLPE is prevalent, it is reasonable to anticipate that the HFLPE on achievement emotions will be consistently negative, even when offsetting the positive effects of selective school advantages. In this study, we focused on an extreme case. Traditionally, studies on the BFLPE and HFLPE compared the academic self-concepts and achievement emotions of students with similar abilities but who attended different types of schools. However, in reality, students who attend selective schools are often more competent than their counterparts in regular schools; in some selective schools in China, the lowest-ranking students are better than the top-ranking students in regular schools in terms of academic performance because in China’s education system, which is highly segregated by achievement, selective schools are usually given priority to enroll the top-ranking students in school entrance exams before regular schools have the chance to begin enrollment. If we compare the achievement emotions of two students whose abilities differ greatly, will the student with higher ability but ranked lower in a selective group be less happy than the student with lower ability but ranked higher in a regular group? Will the positive effects of these objective conditions successfully counterbalance the negative HFLPE resulting from students’ subjective social comparison? To answer these questions, we sought to determine whether the psychological HFLPE on achievement emotions is so overwhelming that it outweighs the physical effects of student abilities and the advantages of selective schools.

## 2. Research Hypotheses

The key to studying the HFLPE lies in exploring the counterbalancing effects of school status and individual ranking on students’ achievement emotions. If students attend a prestigious selective school, their emotional well-being may be improved by their personal gain and that of their family in terms of their reputation, but they may suffer a loss due to the fierce competition with their high-achieving peer group. Whether students’ gain in school status can eclipse their loss in individual ranking was the main question of this study. Therefore, school status and individual ranking were the two main research variables.

### 2.1. School Status

#### 2.1.1. Definition and Relationship with Achievement Emotions

In this study, school status refers to the synthesis of a series of objective school strengths, such as overall reputation, education quality, and student attainment. For our research purposes, we defined whether a school enjoys high status based on social consensus. Specifically, we chose two senior middle schools in Guangdong province, China, where one was a provincial selective school and the other was a municipal regular school. The provincial selective school preferentially enrolls the top-ranking students in the province based on provincial senior middle school entrance exams and has long been widely acknowledged as one of the best schools in all respects nationally; it is commonly known to all Guangdong residents that students who can attend this school have superior academic ability and will bring honor to themselves and their families. The municipal regular school is a mid-to-low-level senior middle school (evaluated by student source or education quality) randomly chosen in the same city, where its top-ranking students lag far behind the bottom-ranking students in the selective school in terms of academic performance in both senior middle school and college entrance exams. According to numerous studies on the BFLPE [14,15,16,17,18,25,26,27,28], the status of the selective school is expected to bring its students more positive and less negative achievement emotions than the status of the municipal school. Hence, we proposed our first hypothesis:

**H1.**  *Higher school status significantly predicts more positive (H1a) and less negative (H1b) achievement emotions.*

#### 2.1.2. Mediators of the Relationship between School Status and Achievement Emotions

As school status is an objective physical indicator, perceived status is the real psychological factor that influences students’ achievement emotions. In this study, perceived school status was measured as students’ sense of school belonging. Abundant evidence [30,31] showed that as a means of impression management, people enjoy “basking” in the reflected glory of distinguished individuals or highly valued social groups; the mere fact of associating with successful individuals produces an identification or assimilation effect on people, which, although illusory, makes them feel as successful as those around them. The BFLPE literature revealed the reflected glory effect of selective schools [25], indicating that students who are surrounded by a group of excellent peers in a high-status school develop more positive academic self-concepts (e.g., confidence) and more positive achievement emotions (e.g., pride) by virtue of attending the school, even more so if the school’s selection is highly valued or the selection process is highly visible (e.g., fair unified exams). Furthermore, academic self-concepts are defined as cognitive representations of one’s academic ability that are stored in long-term memory. They were shown to be positively associated with achievement emotions, with more stable academic self-concepts playing a mediating role in the relationship between a sense of school belonging and achievement emotions [8]. Therefore, we proposed our second to sixth hypotheses:

**H2.**  *Higher school status significantly predicts a higher sense of school belonging.*

**H3.**  *Higher school status significantly predicts better academic self-concepts.*

**H4.**  *A higher sense of school belonging significantly predicts more positive (H4a) and less negative (H4b) achievement emotions.*

**H5.**  *A higher sense of school belonging significantly predicts better academic self-concepts.*

**H6.**  *Better academic self-concepts significantly predict more positive (H6a) and less negative (H6b) achievement emotions.*

**Figure 1:**
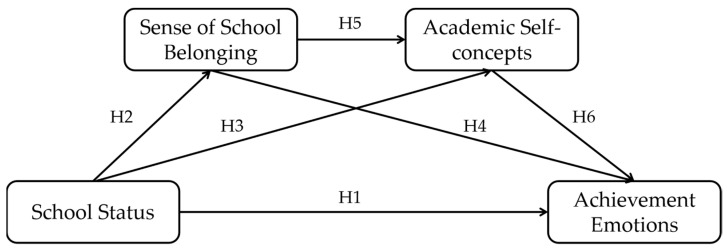
A diagram of our serial mediation model.

The diagram of our serial mediation model shown in Figure 1 summarizes the six hypotheses above.

### 2.2. Individual Ranking: The Moderator

**Figure 2:**
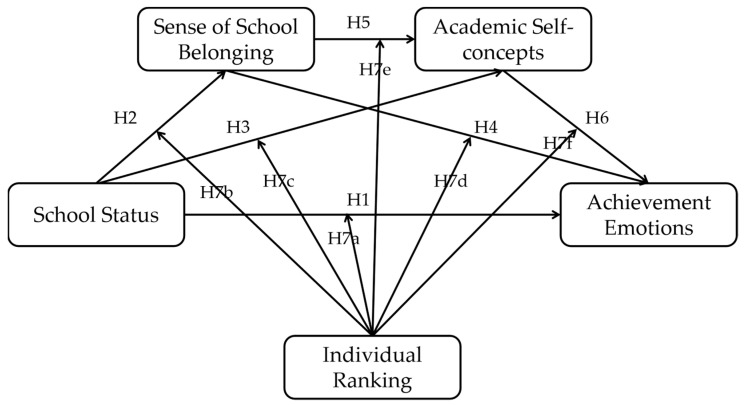
A diagram of our final research model—a moderated serial mediation model.

In most cases, attending a selective school will result in an individual’s low ranking because of increased peer competition, which may lead to more negative and less positive achievement emotions due to negative social comparison. Here, individual ranking is also an objective indicator that is reflected by academic achievement, which is usually positively associated with achievement emotions: the higher the ranking, the more positive and the less negative the achievement emotions. However, when combining the effects of school status and individual ranking on achievement emotions, the two effects may no longer be linear but create an interaction. A study [24] conducted in Chinese Taiwan revealed that class ranking had different effects on students with different learning abilities; for those with low learning ability, a high class ranking was beneficial for their future progress, but for students with high learning ability, a high class ranking was disadvantageous. Similarly, because in our study, attending a selective school represented high academic ability, we hypothesized that individual ranking has a significant moderating effect on the relationship between school status and achievement emotions while moderating other mediating paths. Thus, our seventh hypothesis proposed below can be integrated into the research model in Figure 1 and the final moderated serial mediation model is displayed in Figure 2.

**H7.**  *Individual ranking significantly moderates paths H1 to H6 (H7a–H7f).*

## 3. Methods

### 3.1. Participants

Before recruiting the participants, we used G*Power 3.1 (Düsseldorf, Germany) to estimate the minimum sample size suitable for a 2 × 2 multivariate analysis of variance (MANOVA). When setting the effect size (*f*^2^) at 0.15 (medium level), the error probability (α) at 0.05 (as a common practice), the power (1 − *β*) at 0.8 (as a common practice), the numerator *df* at 1, and the number of groups at 4, the calculated minimum sample size was 128.

In reality, we used a random cluster sampling method to choose six grade 10 classes (around 50 students in each class) from a provincial selective school and a municipal regular school to complete our questionnaire. Based on their average scores on four grade-unified exams in their first year of senior middle school, the students were classified into three groups—top-ranking (25%), middle-ranking (50%), and bottom-ranking (25%) students—by the teacher in charge of their class, and the middle-ranking students were excluded from our analysis. After eliminating invalid questionnaires (passing on more than five items, long strings of invariant responses, or inconsistent responses on similar items), 455 valid questionnaires were retained for our formal analysis. The participants’ basic information is shown in Table 1.

### 3.2. Procedures

The questionnaire survey was conducted at the end of the second semester. After agreeing to participate, the students were divided into class units and asked to complete three psychological scales to measure their sense of school belonging, academic self-concepts, and achievement emotions separately under the supervision of the teacher in charge of their class and a master’s degree student in psychology. The participants were informed that the survey was conducted in a registered manner, that their answers were strictly confidential, and that there was no right or wrong answer to each question. The participants were asked to answer independently according to their actual situation within 10 min, after which the questionnaires were collected immediately. Next, the teacher in charge of the class ranked the students according to their average scores on the four grade-unified exams in their first year.

### 3.3. Variables and Measures

#### 3.3.1. Achievement Emotions

The dependent variable of our study was achievement emotions. To measure achievement emotions, because the AEQ developed by Pekrun and his colleagues [2] is too complicated and does not have a Chinese version, we modified the scale. In the HFLPE study conducted in Germany by Pekrun et al. [14], the valence (positive or negative) of achievement emotions played an important role, as emotions of the same valence showed a similar trend, while emotions of different valences showed opposite trends. Therefore, we took valence as the only dimension of our scale and extracted typical AEQ items [2] with guidance from a psychologist. After a discriminatory analysis, exploratory factor analysis, confirmatory factor analysis (CFA), and reliability analysis, 22 items were formally retained, 11 for positive achievement emotions (e.g., “I can cope with my studies with ease”) and 11 for negative achievement emotions (e.g., “I’m very tired of the learning content”). The participants used a 5-point Likert scale, ranging from 1 (*strongly disagree*) to 5 (*strongly agree*). Cronbach’s α coefficients for the positive achievement subscale, the negative achievement subscale, and the full scale were 0.911, 0.903, and 0.773, respectively, indicating good internal consistency. The CFA results also demonstrated that the scale had acceptable construct validity: *χ*^2^/*df* = 706.082/208 = 3.395, RMSEA = 0.067, SRMR = 0.059, CFI = 0.912, and TLI = 0.902.

#### 3.3.2. School Status

School status was a dichotomous independent variable and was defined by the researchers based on a social consensus of the overall reputation, student attainment, and education quality, among others. The provincial selective school was coded 1 and the municipal regular school was coded 0. It should be noted that even the lowest-ranking students in the selective school were far more academically competent than the highest-ranking students in the regular school.

#### 3.3.3. Individual Ranking

Individual ranking was another dichotomous independent variable and a moderating variable and was grouped into top ranking (25%), middle ranking (50%), and bottom ranking (25%) by the teachers according to their students’ average performance in their first year of senior middle school. It was coded 1 for top ranking and 0 for bottom ranking, while the middle-ranking students were excluded from our formal analysis.

#### 3.3.4. Sense of School Belonging

Sense of school belonging was one of the mediating variables in the relationship between individual ranking and achievement emotions tested in this study. To measure the students’ sense of school belonging, we developed a 6-item Chinese scale based on the collective self-esteem scale developed by Luhtanen and Crocker, which has often been used to assess in-group identification [32]. The six items belonged to one dimension; a sample item is “I feel strongly that I am a member of the school”. The response format was a 9-point Likert scale ranging from 1 (*strongly disagree*) to 9 (*strongly agree*). Cronbach’s α was 0.953 and the fit indexes of the model in the CFA were *χ*^2^/*df* = 172.364/9 = 19.152, SRMR = 0.028, CFI = 0.952, and TLI = 0.921.

#### 3.3.5. Academic Self-Concept

Academic self-concept was another mediating variable in the relationship between individual ranking and achievement emotions tested in the study. To measure the students’ academic self-concepts, we relied on the Adolescent Students’ General Academic Self-Concept Scale developed by Guo et al. [33] and developed a short version of the scale, containing only one dimension and six items (e.g., “In general, I am a gifted student”) measured on a 5-point Likert scale, ranging from 1 (*strongly disagree*) to 5 (*strongly agree*). Cronbach’s α was 0.897 and the fit indexes of the model in the CFA were *χ*^2^/*df* = 96.303/9 = 10.7, SRMR = 0.035, CFI = 0.953, and TLI = 0.922.

### 3.4. Testing for Common Method Bias

As more than one scale was used in our study, to avoid common method bias, we conducted Harman’s single-factor test. The results showed that the percentage of variance explained by the first common factor was 26.506% (less than 40%), indicating that common method bias was not a problem in our study.

### 3.5. Statistical Analysis

SPSS 24.0 (Chicago, IL, USA) was used for data analysis. First, we present the overall picture of the students’ achievement emotions and other variables using descriptive statistics and correlation analysis. We also examined gender differences between the variables.

Second, we compared the students’ achievement emotions in terms of school status and individual ranking between the two schools using a 2 × 2 MANOVA with non-repeated measures. As exploring the counterbalancing effects of school status and individual ranking was the key to studying the HFLPE, we focused specifically on comparing achievement emotions between the bottom-ranking students in the selective school and the top-ranking students in the regular school. If the emotional well-being of the bottom-ranking students in the selective school was worse than that of the top-ranking students in the regular school, it would show that the HFLPE is valid in the Chinese collectivist context and is so strong that it even exists in the extreme case used in our study. In addition, we used an analysis of variance (ANOVA) to test whether there was an interaction between school status and individual ranking to verify H7.

Finally, we conducted a path analysis to explore the mediating effects of the sense of school belonging and academic self-concepts on the relationship between school status and achievement emotions (H1–H6) to test the full research model (Figure 2).

## 4. Results

### 4.1. Preliminary Analysis

The descriptive statistics and Pearson correlation coefficients of the study variables are presented in Table 2. In general, the correlation coefficients were as expected for H1b, H2, H3, H4, H5, and H6.

As a study found that there were gender differences in achievement emotions between Chinese adolescent students [34], we examined gender differences in our six study variables (see Table 3). The results showed that the girls in our sample had significantly lower positive academic self-concepts and achievement emotions than the boys, indicating that gender should be controlled for in the following analysis.

### 4.2. The 2 × 2 ANOVA

We used a 2 (school status: selective vs. regular) × 2 (individual ranking: top vs. bottom) MANOVA with gender as a controlled covariate to test whether the HFLPE on positive and negative achievement emotions exists in the Chinese collectivist context.

For positive achievement emotions, the main effect of school status was not significant (*F* [1450] = 0.158, *p* = 0.692), while the main effect of individual ranking was significant (*F* [1450] = 33.198, *p <* 0.001, partial *η*^2^ = 0.069). In addition, their interaction was significant (*F* [1450] = 14.468, *p <* 0.001, partial *η*^2^ = 0.031).

For negative achievement emotions, the main effects of school status (*F* [1454] = 21.607, *p <* 0.001, partial *η*^2^ = 0.045) and individual ranking (*F* [1454] = 38.446, *p* < 0.001, partial *η*^2^ = 0.078) were significant. However, their interaction was not significant (*F* [1454] = 0.154, *p =* 0.695).

The results of Table 4 revealed a surprising phenomenon: the bottom-ranking students in the selective school had significantly lower positive achievement emotions than the top-ranking (*t* = −4.189, *df* = 222, *p <* 0.001) and bottom-ranking (*t* = −2.004, *df* = 195.593, *p =* 0.046) students in the regular school and slightly more negative achievement emotions than the top-ranking students in the regular school (*t* = 1.082, *df* = 222, *p* = 0.280), although the result was not significant; however, the top-ranking students in the selective school had significantly more positive achievement emotions than the top-ranking (*t* = 2.322, *df* = 256, *p =* 0.021) and bottom-ranking (*t* = 3.621, *df* = 232, *p <* 0.001) students in the regular school. These results showed that the positive achievement emotions of top-ranking and bottom-ranking students differed strongly in the selective school but slightly in the regular school, indicating that the environment of fierce peer competition in selective schools can increase the gap between individual rankings, and thus, cause low-ranking students to experience intense psychological discomfort.

The results also showed that the students’ achievement emotions were more influenced by subjective social comparison than by objective evaluation; that is, the HFLPE existed in the Chinese collectivist culture, even in the extreme case where the bottom-ranking students in selective schools far outperformed the top-ranking students in regular schools on all fronts.

### 4.3. Testing the Research Model

The results reported in Section 4.2 demonstrated that school status and individual ranking moderated each other’s effect on positive achievement emotions. Next, we conducted a path analysis to further test the mediating effects of the sense of school belonging and academic self-concepts to test the full research model (see Figure 1).

We applied the SPSS macro Model 92 to test H1–H7, with positive and negative achievement emotions as dependent variables and controlling for gender as a covariate.

**Figure 3:**
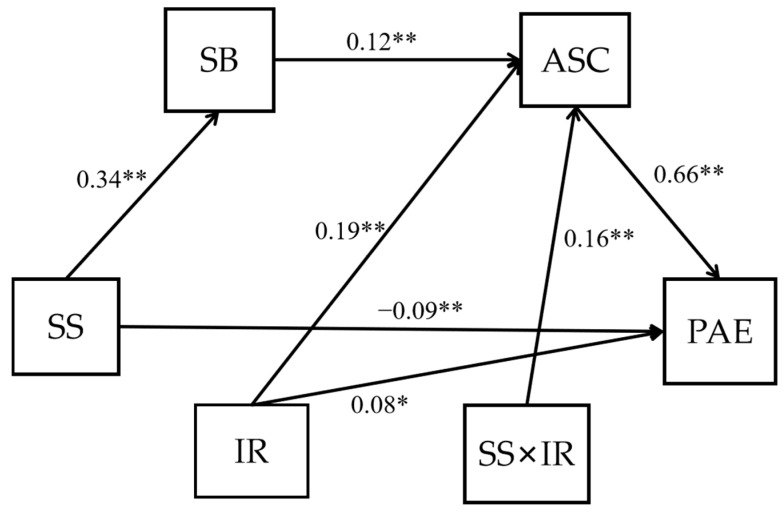
A diagram of the moderated serial mediation model, with positive achievement emotions as the dependent variable. SS—school status; SB—sense of school belonging; ASC—academic self-concepts; PAE—positive achievement emotions; IR—individual ranking; * *p* < 0.05; ** *p* < 0.01.

**Figure 4:**
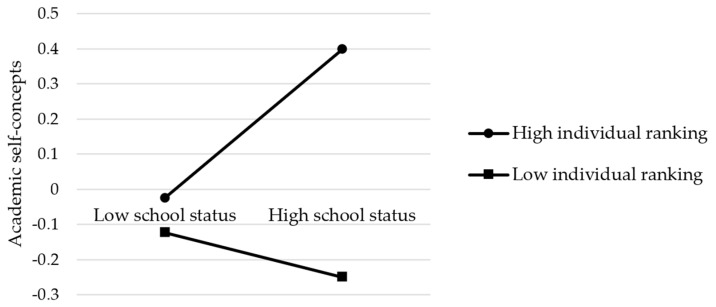
A diagram of the moderating effect of individual ranking on the path from school status to academic self-concepts.

For positive achievement emotions (see Figure 3 and Table 5), the results showed that the effect of school status on positive achievement emotions was serially mediated by the students’ sense of school belonging and academic self-concepts. In addition, individual ranking significantly moderated the relationship between school status and academic self-concepts alone and in interaction with school status, whereas school status had no effect on academic self-concepts. Therefore, H1a, H2, H3, H5, H6a, and H7c were verified. Next, we tested the moderated path mechanism using a simple slopes analysis to determine how the effect of school status on positive achievement emotions differs at different levels of individual ranking. As Figure 4 shows, individual ranking played a key role in affecting academic self-concepts because the top-ranking students had more positive academic self-concepts than their lower-ranking counterparts, regardless of school status. In addition, when the individual ranking was low, school status had a negative influence on academic self-concepts. These results suggested that the BFLPE is so strong that it also existed in the extreme case studied here.

**Figure 5:**
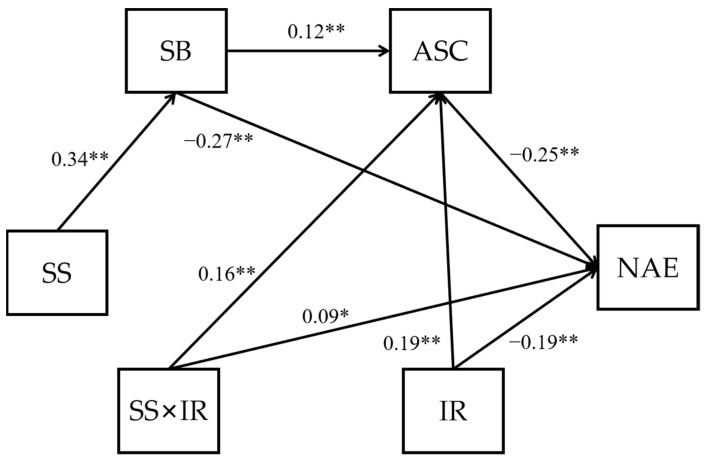
A diagram of the moderated serial mediation model, with negative achievement emotions as the dependent variable. SS—school status; SB—sense of school belonging; ASC—academic self-concepts; PAE—positive achievement emotions; IR—individual ranking; * *p* < 0.05; ** *p* < 0.01.

**Figure 6:**
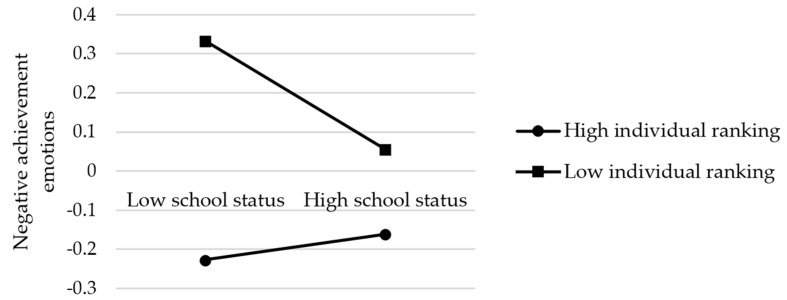
The diagram of the moderating effect of individual ranking on the path from school status to negative achievement emotions.

For negative achievement emotions (see Figure 5 and Table 6), the results showed that the effect of school status on negative achievement emotions was also serially mediated by the students’ sense of school belonging and academic self-concepts. However, unlike the model of positive achievement emotions (Figure 3), the path from school status to negative achievement emotions was only mediated by the students’ sense of school belonging. In addition, individual ranking had a significant moderating effect on both academic self-concepts and negative achievement emotions, which was in line with the ANOVA results reported in Table 4. As shown more clearly in Figure 6, the simple slopes analysis revealed that individual ranking also played a key role in affecting negative achievement emotions because the bottom-ranking students had more negative emotions than the top-ranking students regardless of school type. Furthermore, consistent with our results for academic self-concepts, when the individual ranking was low, high school status negatively affected the students’ emotional well-being. Therefore, H1b, H2, H3, H4, H5, H6b, H7a, and H7c were verified.

## 5. Discussion

The literature widely acknowledges the BFLPE and HFLPE, and our study provides further evidence for this field with a different methodology. Traditional studies on the BFLPE and HFLPE mostly drew research findings from hierarchical linear modeling of a large sample of data covering schools at all levels and students in all ranks and compared academic self-concepts and achievement emotions of the students of similar abilities. Our study, however, only focused on extreme conditions. One extreme condition was that we selected two schools (one selective school and one regular school) where even the bottom-ranking students in the former school enjoyed full-spectrum dominance in terms of objective academic competence over the top-ranking students in the latter school. The other extreme condition was that our study was conducted in the Chinese collectivist cultural setting where people are believed to be more obsessive with the glory of selective schools. These two extreme conditions served to maximize the positive reflected-glory effect of being in a selective school and minimize the negative social comparison contrast effects that result from being ranked low in the school. Surprisingly, the results still demonstrated strong BFLPE and HFLPE by showing that the bottom-ranking students in the selective school had lower positive academic self-concepts and more negative achievement emotions than their top-ranking counterparts in the regular school. This finding evidently suggested that students relied more on social comparison than on objective self-evaluation standards and that individual ranking played a key role in affecting students’ emotional well-being and an even more important role in a more competitive environment.

This study provided stronger evidence for the literature on the BFLPE and HFLPE and broad areas of social comparison theory. The results should also be of interest to policymakers, administrators, coaches, and teachers from a practical perspective. Traditional BFLPE and HFLPE research focused primarily on their important and surprising implications for the school placement of gifted students, neglecting the assumed advantages of attending selective schools because of the emotional costs incurred; that is, if a candidate barely manages to be admitted to a highly selective school and will be ranked low in that school, it would be better for that student to be a top-ranking student in a regular school in terms of mental well-being. However, given our findings that even the most talented students who enjoy the absolute advantages of their academic competence among their peers will easily become frustrated when confronted with a smarter peer, the negative effects of social comparison seem inevitable as long as individual differences exist at the group, class, or school level. Even if policymakers establish systems with lower levels of achievement-based stratification, as suggested by Pekrun and his colleagues [14], this may not reduce the negative emotional consequences of being inferior in terms of academic performance. Therefore, greater importance should be given to mental health education in schools.

First, special attention should be given to underachievers at the group, class, and school levels. As was also suggested by the PISA data, in every school around the world, there are students in very good mental condition, but there are also many students who experience worrying psychological problems [35]. Thus, school administrators and teachers are expected to average test rankings, help students rationally understand their exam results by identifying their knowledge gaps and weaknesses, place more emphasis on intrapersonal mastery over competitive goals [36], provide timely psychological counseling for students with poor exam results, and implement the necessary systems to monitor and intervene in individual psychological crises [37].

In addition, freshmen newly admitted to a school should be given special consideration, as they are more likely to encounter difficulties in adapting to their new environment and to feel lost, which will increase their anxiety. Studies showed that Chinese adolescents are vulnerable to psychological derailments when they start a new period of school life (e.g., entering senior high school or college), which may lead to mental health problems, such as depression [38]. Investigations of the academic burden of Chinese students also suggested that during transition periods of school stages, students are more sensitive to the school workload, and thus, need more guidance on psychological adjustment and pressure management [39]. According to Pekrun et al. [14] and our study, students’ achievement emotions are easily affected by their reference group, the sudden change of which will make the effects of social comparison even more visible, leading to self-doubt and the denial of previous concepts. One suggestion for schools is to establish special educational programs, such as team-building or military training activities, during summer vacation to alleviate freshmen’s maladjustment problems.

## 6. Limitations

Nevertheless, our study had some limitations. First and foremost, as there were only two schools sampled, it may not be possible to fully replicate such a strong BFLPE and HFLPE identified in our study in other schools, i.e., the conclusions of our study may not be well generalizable to other cases. However, this does not diminish the value of our paper as a case report in highlighting the role of social comparison in self-evaluation by providing stronger evidence than previous research. As the BFLPE and HFLPE have been frequently examined by numerous studies and proved robust by meta-analyses, selecting other schools as the sample would not result in completely different results but only slightly different effects that are still in line with the social comparison theory. For future case studies, samples from selective high schools in China’s provinces other than Guangdong should be recruited because there are very few selective high schools in each province where even the bottom-ranking students still outperform the students in regular schools. Second, due to some ethical restrictions in the student survey set by the sampled schools, some confounding variables, e.g., economic, social, and cultural status, were not able to be measured and controlled for in this study, which might also limit the generalizability of the research conclusions. Third, we only examined the achievement emotions of grade 10 students, who had attended their school for less than a year; therefore, we could not rule out the possibility that such strong effects were partially the result of their maladjustment to their new environment and reference group. Thus, whether the effects we observed will last over time should be further investigated using a longitudinal design.

## 7. Conclusions

In this study, we reexamined the HFLPE on achievement emotions in a previously unexplored extreme case in the Chinese collectivist context. The results surprisingly demonstrated that the HFLPE was so strong that the emotional well-being of the bottom-ranking students in the selective school was lower than that of the top-ranking students in the regular school, even though the academic abilities of the bottom-ranking students far exceeded those of the top-ranking students, indicating that students relied more on subjective social comparison than on objective self-evaluation standards. Overall, this study revealed the key role of individual ranking in affecting students’ emotional well-being, enriched the research on academic self-concepts and achievement emotions, presented strong evidence for broad areas of social comparison theory, and has important implications for educational practices such as school placement and mental health education.

## Figures and Tables

**Table 1:** Basic information of the valid participants.

School Status	Top-Ranking			Bottom-Ranking			Total
	Boys	Girls	Total	Boys	Girls	Total	
Selective School	50	80	130	56	38	94	224
Regular School	57	70	127	36	68	104	231
Total	107	150	257	92	106	198	455

**Table 2:** Descriptive statistics and Pearson correlation coefficients for the study variables.

	*M*	*SD*	1	2	3	4	5	6
1. School status	0.493	0.501	1					
2. Individual ranking	0.563	0.497	0.024	1				
3. Sense of school belonging	7.063	1.53	0.417 **	0.038	1			
4. Academic self-concepts	2.868	0.67	0.141 **	0.250 **	0.149 **	1		
5. Positive achievement emotions	2.697	0.693	0.024	0.244 **	0.124 **	0.652 **	1	
6. Negative achievement emotions	2.287	0.713	−0.211 **	−0.278 **	−0.319 **	−0.285 **	−0.261 **	1

** *p* < 0.01.

**Table 3:** Gender differences in the study variables.

	*M* ± *SD*		*t*	*p*
	Girls (*n* = 256)	Boys (*n* = 199)		
School status	0.46 ± 0.50	0.53 ± 0.50	−1.519	0.130
Individual ranking	0.59 ± 0.49	0.54 ± 0.50	1.029	0.304
Sense of school belonging	7.18 ± 1.43	6.90 ± 1.65	1.890	0.059
Academic self-concepts	2.74 ± 0.65	3.05 ± 0.65	−5.109	0.000 **
Positive achievement emotions	2.59 ± 0.67	2.84 ± 0.70	−3.859	0.000 **
Negative achievement emotions	2.28 ± 0.73	2.30 ± 0.70	−0.341	0.733

** *p* < 0.01.

**Table 4:** ANOVA results for positive and negative achievement emotions.

	Positive Achievement Emotions		Negative Achievement Emotions	
	Top-Ranking	Bottom-Ranking	Top-Ranking	Bottom-Ranking
Selective School				
*M*	2.94	2.41	1.98	2.35
*SD*	0.71	0.61	0.68	0.65
*N*	130	94	130	96
Regular School				
*M*	2.75	2.60	2.25	2.67
*SD*	0.61	0.73	0.69	0.65
*N*	127	104	128	104

**Table 5:** Moderated serial mediation results, with positive achievement emotions as the dependent variable. SS—school status; SB—sense of school belonging; ASC—academic self-concepts; PAE—positive achievement emotions.

Mediation Path	Mediating Variable	Effect	Boot SE	95% Confidence Interval	
				Boot LLCL	Boot ULCL
SS → ASC → PAE	Low individual ranking	−0.083	0.034	−0.152	−0.016
	High individual ranking	0.130	0.033	0.069	0.196
	Difference	0.213	0.048	0.121	0.307
SS → SB → ASC → PAE	Low individual ranking	0.017	0.015	−0.012	0.048
	High individual ranking	0.039	0.016	0.011	0.072
	Difference	0.022	0.022	−0.020	0.067

**Table 6:** Moderated serial mediation results, with negative achievement emotions as the dependent variable. SS—school status; SB—sense of school belonging; ASC—academic self-concepts; NAE—negative achievement emotions.

Path	Mediating Variable	Effect	Boot SE	95% Confidence Interval	
				Boot LLCL	Boot ULCL
SS → SB → NAE	Low individual ranking	−0.064	0.030	−0.132	−0.013
	High individual ranking	−0.116	0.030	−0.179	−0.064
	Difference	−0.052	0.042	−0.134	0.033
SS → ASC → NAE	Low individual ranking	0.020	0.014	−0.001	0.052
	High individual ranking	−0.061	0.022	−0.110	−0.025
	Difference	−0.081	0.026	−0.139	−0.037
SS → SB → ASC → NAE	Low individual ranking	−0.004	0.005	−0.016	0.003
	High individual ranking	−0.018	0.008	−0.035	−0.005
	Difference	−0.014	0.009	−0.032	0.003

## Data Availability

The data presented in this study are available from the corresponding author upon request due to privacy and ethical restrictions.
